# CYP39A1 polymorphism is associated with toxicity during intensive induction chemotherapy in patients with advanced head and neck cancer

**DOI:** 10.1186/s12885-015-1776-x

**Published:** 2015-10-16

**Authors:** Thomas Melchardt, Clemens Hufnagl, Teresa Magnes, Lukas Weiss, Georg Hutarew, Daniel Neureiter, Alexander Schlattau, Gerhard Moser, Alexander Gaggl, Wolfgang Tränkenschuh, Richard Greil, Alexander Egle

**Affiliations:** 1 Department of Internal Medicine III, Paracelsus Medical University Salzburg, Müllner-Hauptstrasse 48-5020, Salzburg, Austria; 2 Salzburg Cancer Research Institute (SCRI), Müllner-Hauptstrasse 48-5020, Salzburg, Austria; 3 Cancer Cluster Salzburg (CCS), Müllner-Hauptstrasse 48-5020, Salzburg, Austria; 4 Institute of Pathology, Paracelsus Medical University Salzburg, Müllner-Hauptstrasse 48-5020, Salzburg, Austria; 5 Institute of Radiology, Paracelsus Medical University Salzburg, Müllner-Hauptstrasse 48-5020, Salzburg, Austria; 6 Department of Otorhinolaryngology, Paracelsus Medical University Salzburg, Müllner-Hauptstrasse 48-5020, Salzburg, Austria; 7 Department of Oral and Maxillofacial Surgery, Paracelsus Medical University Salzburg, Müllner-Hauptstrasse 48-5020, Salzburg, Austria

**Keywords:** SNP, Docetaxel, Head and neck cancer, Induction chemotherapy, Toxicity

## Abstract

**Background:**

Induction chemotherapy incorporating docetaxel, cisplatin and 5- fluorouracil before radiotherapy may improve the outcome of patients with advanced head and neck cancer. Nevertheless, the addition of docetaxel increases hematological toxicity and infectious complications. Therefore, genetic markers predicting toxicity and efficacy of this treatment regimen may help to identify patients, who would have the most benefit from this intensive treatment.

**Methods:**

A cohort of 78 patients with advanced head and neck cancer treated with induction chemotherapy was assessed for clinical outcome and toxicity during treatment with curative intention. Genetic polymorphisms primary associated with treatment efficacy (ERCC2-rs13181, rs1799793, ERCC1-rs3212986, rs11615, XRCC1-rs25487) or with docetaxel caused toxicity (CYP39A1-rs7761731, SLCO1B3-rs11045585) were evaluated in all patients. The results of these analyses were correlated with the clinical outcome of the patients (loco regional control, progression free survival, overall survival) and treatment related toxicity during induction chemotherapy.

**Results:**

Median progression free survival and overall survival was 20 and 31 months in an intention to treat analysis, respectively. Overall response rate to induction chemotherapy was high with 78.1 % of all patients. None of the polymorphisms tested was associated with the clinical outcome of the patients. Genotype A of the CYP39A1 rs7761731 polymorphism was associated with a higher incidence of leucopenia and infections or death during induction chemotherapy.

**Conclusions:**

Intensive induction chemotherapy results in a high response rate in the majority of patients. None of the polymorphisms tested was associated with the clinical outcome of the patients. The CYP39A1 polymorphism rs7761731 may help to identify patients at high risk for treatment related toxicity.

## Background

The management of patients with advanced head and neck cancer is a complex process requiring a multidisciplinary approach including effective cytotoxic regimens, radical surgery and radiotherapy (RT) [1]. It has been shown that patients with locally advanced disease not suitable for surgery can be cured with induction chemotherapy followed by definitive radiochemotherapy (RCTX) [2]. Two randomized controlled phase III trials showed an overall survival (OS) benefit for the addition of docetaxel to induction chemotherapy with a platinum compound and 5-fluorouracil (TPF) followed by RT with or without low-dose carboplatin [3, 4]. Therefore, this approach can be used in patients with locally advanced disease not suitable for surgery although two recently published trials doubt the clinical value of induction treatment in comparison to primary RCTX [5, 6]. Furthermore, the specific value of radio-sensitizing therapy in addition to RT after TPF is unclear.

This intensive treatment strategy is also associated with significant toxicity. Furthermore, some patients with advanced age and severe comorbidities often caused by substance abuse do not qualify for this treatment. Besides such clinical risk factors, single nucleotide polymorphisms (SNPs) of genes influencing the mode of action or the metabolism of cytotoxic drugs may identify patients, who on the one hand have more benefit from intensive treatment or on the other hand are at higher risk for toxicity. Three SNPs of the DNA repair genes ERCC1, ERCC2 and XRCC1 influencing the efficacy of cisplatin have been described to predict better outcome in a heterogeneous cohort of 103 patients with head and neck cancer treated with cisplatin during induction chemotherapy or RT [7]. Nevertheless, this report included only 40 patients treated with modern induction chemotherapy with cisplatin, fluoropyrimidine and taxanes. Furthermore, data on the influence of genetic polymorphisms on the incidence of treatment related toxicities are still missing. Docetaxel-induced toxicity, especially bone marrow suppression, is reported to be influenced by such polymorphisms [8–11].

To test the value of these polymorphisms for predicting the efficacy and toxicity of the TPF regimen we assessed the clinical outcome and the genetic signature of 6 genes in all patients treated with TPF therapy for advanced head and neck cancer since 2006 at our cancer center.

## Methods

### Patients

All patients included for this analysis were diagnosed with advanced head and neck cancer between January 2006 and December 2013 and consecutively treated with TPF chemotherapy as first line therapy treatment at the 3rd Medical Department of the Paracelsus Medical University Salzburg. Follow-up data were available for all patients at the last update of the database on the 28th of February 2014. Clinical data were also obtained by telephone interviews of the general practitioners or relatives if needed. Three cycles of induction treatment including docetaxel (75 mg/m^2^), cisplatin (75 mg/m^2^) and 5-fluorouracil (750 mg/m^2^ continously for 5 days) were planned in all patients in analogy to the TAX 323 trial [4] as our local standard. Primary use of granulocyte-colony stimulating factor support was mandatory and all patients were hospitalized at least for the first cycle of treatment until bone marrow recovery. Prophylactic antibiotic treatment using a fluoroquinolone was also an institutional practice. Response to induction chemotherapy was clinically assessed before each cycle and radiologic response was assessed after 2 or 3 cylces with the method, which was used for primary staging (magnetic resonance imaging in 82 % of all cases).

After completion of induction chemotherapy all patients were referred for concomitant radiotherapy in combination systemic treatment (cisplatin, carboplatin cetuximab). Patients without response to induction chemotherapy after two cycles were referred for salvage surgery or immediate radiotherapy if unresectable.

This retrospective study was approved by the Ethics Committee of the provincial government of Salzburg, Austria (415-EP/73/340-2014) and written informed consent was obtained from all patients. Clinical data including the stage of disease, loco regional control (LRC), OS and progression free survival (PFS) were analyzed by chart based review. PFS was calculated from start of therapy until disease progression, diagnosis of another tumor or death from any cause. Common toxicity criteria for adverse events version 4.0 (CTCAE 4.0) were used to assess treatment related toxicity.

Statistical analyses were performed using IBM® SPSS® statistics software, version 20. Mann–Whitney-U-test and Pearson’s chi-squared test were used for univariate analyses, where appropriate. Survival was estimated using Kaplan-Meier curve analysis, with statistical comparison using the log-rank statistic. A two-tailed significance-level of 0.05 was considered statistically significant. Only statistically significant factors were included into multivariate Cox-regression analyses. Due to the exploratory and hypothesis generating design of the present SNP data study no adjustment for multiple testing was applied [12].

### SNP genotyping

SNPs influencing the pharmacodynamics of cytotoxic agents were chosen for analysis after extensive review of the available literature dealing with the metabolism of drugs within the TPF regimen. The following SNPs were selected because of reported effect on drug efficacy: ERCC2-rs13181 and rs1799793, ERCC1-rs3212986 and rs11615, XRCC1-rs25487 [7, 13]. The CYP39A1-rs7761731 and SLCO1B3 rs11045585 polymorphisms are reported to be associated with treatment related toxicity caused by docetaxel, which is the major component of hematological toxicity in the used induction chemotherapy regimen [8–11].

Germ line DNA was extracted from frozen peripheral blood cells in 65 % of the patients. In the other patients without frozen peripheral blood cells we used extracted germ line DNA from diagnostic formalin fixed tissue samples with no histologically observed tumor infiltration. This procedure was performed using the Maxwell 16 FFPE Plus LEV or Maxwell Blood DNA Purification KIT. Extracted DNA was amplified using the Genotyping Master Mix and SNP Genotyping assay from TaqMan®. Afterwards allelic discrimination was performed using the software of the Applied Bio System 7500 system (Thermo Fischer). The assessed genotypes were correlated and then subjected to correlative analyses with the clinical outcome of the patients (LCR, PFS, OS) and treatment related toxicity during induction chemotherapy.

## Results

### Patient characteristics

All 78 patients recruited in the study were consecutively treated with TPF induction chemotherapy for advanced head and neck cancer between 2006 and 2013 (see Table 1). In detail, all patients were Caucasians with a median age of 56.0 years and 88.5 % of the patients were male. As expected, the majority of the patients (80.7 %) had a primary tumor of the pharynx, 15.4 % were diagnosed with a tumor of the oral cavity. Squamous cell carcinoma was diagnosed in 98.7 % of the patients and all except for one were HIV negative. Median follow-up for alive patients was 56 months.

**Table 1:** Patients characteristics treated with first line induction TPF chemotherapy

	Overall
	*n = 78*
Age (years)	
Median	56
Range	40–72
>60 years (%)	28.2
Sex	
Male	*n* = 69 (88.5 %)
Female	*n* = 9 (11.5 %)
Treatment after TPF (%)	
RT	*n* = 69 (88.5 %)
OP	*n* = 4 (5.1 %)
no further treatment due to toxicity	*n* = 5 (6.4 %)
Nodal stage cN2c or cN3	
Yes	*n* = 24 (30.8 %)
No	*n* = 54 (69.2 %)
cT4 stage (%)	
Yes	*n* = 53 (67.9 %)
No	*n* = 25 (32.1)
Stage of disease	
AJCC Stage 4	*n* = 73 (93.4 %)
AJCC Stage 3	*n* = 5 (6.4 %)
Localisation of primary tumor (%)	
Oral cavity	*n* = 12 (15.4 %)
Pharynx	*n* = 63 (80.7 %)
Paranasal sinus	*n* = 2 (2.6 %)
Not available	*n* = 1 (1.3 %)

*TPF* docetaxel, 5-flurouracil, cisplatin

*CUP* carcinoma of unknown primary

*RT* radiotherapy

*n* number

*AJCC* American Joint Committee on Cancer

### Clinical outcome in patients treated with TPF

**Fig. 1:**
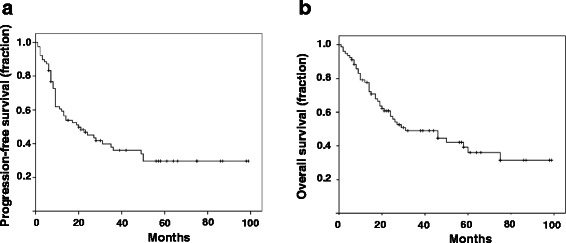
Clinical outcome in patients treated with induction chemotherapy. PFS (median 20 months **a**) and OS (median 31 months **b**) in all patients treated with first line TPF induction chemotherapy

The median PFS and OS of all patients treated with at least 1 cycle of TPF were 20 and 31 months, respectively (see Fig. 1). Overall response rate (ORR) after chemotherapy was 78.1 % including partial remission (PR) in 63 % and complete response (CR) in 15 % of the patients; 13.7 % had stable disease and 8.2 % of the 73 evaluable patients had refractory disease following induction chemotherapy. The therapy following TPF was RT in 88.5 %, surgery of the primary tumor in 5.1 and 6.4 % of the patients received no further treatment due to toxicity or progression (see Table 1). As expected, the presence of AJCC stage 4 disease or a cT4 primary tumor had negative influence on the clinical outcome of the patients (median PFS not reached vs. 17 months *p* = 0.026; 50 vs. 13 months *p* = 0.045), respectively. Nevertheless, multivariate analyses failed to show an independent value of one of these factors, respectively (*p* = 0.263; *p* = 0.116).

Sixty-eight of these 69 patients receiving RT after TPF were treated with a concomitant regimen during RT. As concomitant therapy for RCTX, high dose cisplatin, carboplatin and cetuximab were used in 67.7, 14.7 and 17.6 % of the patients.

Concomitant cisplatin treatment during RT was associated with better LRC (49 vs 11 months *p* < 0.001), PFS (36 vs 9 months *p* < 0.001) and OS (60 vs 19 months *p* = 0.005) than treatment with another drug regimen. To evaluate a possible influence of the previous response to induction therapy on the choice of concomitant treatment we also evaluated only patients with a response to TPF. Concomitant cisplatin treatment was also associated with a better LRC (59 vs 18 months *p* = 0.022) and better PFS (49 vs 14 months *p* = 0.024) in these patients, but OS did not reach statistical significance (60 vs 27 months *p* = 0.094).

### SNP analysis in patients treated with induction chemotherapy

Germ line DNA for analysis of the 6 SNPs rs13181, rs1799793, rs3212986, rs11615, rs25487, rs7761731 were available for all patients treated with TPF chemotherapy. Despite the limited size of the cohort all SNPs were present at the predicted Hardy-Weinberg equilibrium and the allele frequencies were in line with the published databases (see Table 2). None of the SNPs especially those, who were previously described to be relevant for treatment efficacy, had a significant influence on LRC, PFS or OS in our cohort. We also could not observe any difference in the ORR.

**Table 2:** Allele frequency

SNP	n	Genotype frequency			HWE (p)
rs13181 ERCC2	78	19 GG (24.4 %)	35 GT (44.9 %)	24 TT (30.8 %)	0.53
rs3212986 ERCC1	78	10 AA (12.8 %)	24 AC (30.8 %)	44 CC (56.4 %)	0.72
rs 11615 ERCC1	78	27 AA (34.6 %)	36 AG (46.2 %)	15 GG (19.2 %)	0.58
rs 1799793 ERCC2	78	25 CC (32.1 %)	40 AG CT (51.3 %)	13 TT (16.7 %)	0.58
rs 7761731 CYP39A1	77	33 AA (42.9 %)	36 AT (46.8 %)	8 TT (10.4 %)	0.66
rs 25487 XRCC1	78	36 CC (46.2 %)	36 CT (46.2 %)	6 TT (7.7 %)	0.69
rs11045585 SLCO1B3	77	49 AA (63.8 %)	26 AG (33.3 %)	2 GG (2.6 %)	0.50

### Toxicity in patients treated with induction chemotherapy

Overall, 204 cycles of TPF were administered in 78 patients with 73.1 % of patients receiving all of the three planned cycles of therapy. Nevertheless, due to excessive toxicity 11.5 % of the patients received only one cycle of therapy. Therapy related complications (myocardial infarction, 2 septic complications) resulted in 3 deaths during induction chemotherapy (treatment related mortality: 3.8 %). Furthermore, one patient committed suicide after the first cycle of therapy and one patient developed a progressive confusional state after the first cycle of treatment and successful bone marrow recovery without infectious complication and died 2 months later (see Tables 3 and 4). A grade 3 or 4 leucopenia after the first cycle of treatment was observed in 44.7 % of the 76 evaluable patients, but none of them experienced grade 3 or 4 thrombopenia. Grade 3 or 4 infection was observed after 28 of 202 (13.8 %) evaluable cycles of induction therapy and in 22 of 76 (28.9 %) evaluable patients. Renal toxicity higher than grade 1 was documented in 9 out of 202 (4.4 %) cycles in 9 different patients (see also Tables 3 and 4).

**Table 3:** Toxicity during first cycle of induction chemotherapy according to CTCAE 4.0 (76 evaluable patients) by genotype (% per group)

	Genotype rs7761731			Total population (*n* = 76)
	AA (*n* = 33	AT (*n* = 36)	TT (*n* = 8)	
Leucopenia G3/4 (%)	62.5 %	37.1 %	12.5 %	44.7 %
Infection G3/4 (%)	28.1 %	20.0 %	25.0 %	23.1 %
Renal Toxicity > G1 (%)	3.1 %	11.4 %	12.5 %	9.2 %
Thrombopenia G3 (%)	na.	na.	na.	0 %

**Table 4:** Toxicity during induction chemotherapy according to CTCAE 4.0 (76 evaluable patients) by genotype (% per group)

	Genotype rs7761731			Total population (*n* = 76)
	AA (*n* = 33	AT (*n* = 36)	TT (*n* = 8)	
Leucopenia G3/4 (%)	65.6 %	45.7 %	37.5 %	52.6 %
Infection G3/4 (%)	40.6 %	20.0 %	25.0 %	28.9 %
Renal Toxicity > G1 (%)	6.3 %	14.3 %	12.5 %	11.8 %
Thrombopenia G3 (%)	na.	2.8	na.	1.3 %

*na.* not available

Beside the tested polymorphisms associated with drug efficacy described above we also tested two polymorphisms related to docetaxel related toxicity.

The rs7761731 SNP of the CYP39A1 gene is reported to be associated with a higher rate of leucopenia in patients treated with docetaxel [8]. Therefore, we evaluated its influence on the frequency of leucopenia in our patients. Homozygosity of allele A, T and heterozygosity were observed in 42.9, 10.4 and 46.8 % of the patients, respectively (see Table 2). As expected, this polymorphism had no influence on the clinical outcome of patients, Patients with homozygosity for allele A had a significantly higher rate of grade 3 or 4 leucopenia during the first cycle compared to patients with genotype AT and TT (62.5 % vs 32.5 % *p* = 0.01). This negative effects of allele A also persists comparing the frequency of leucopenia during the first cycle with patients with genotype AA and AT to patients with genotype TT (49.2 % vs 12.5 % *p* = 0.048). We also observed a higher rate of a combined endpoint of infections or death during induction chemotherapy in patients with homozygosity for allele A compared to patients with genotype AT and TT (45.4 % vs 22.7 % *p* = 0.035).

Furthermore, we evaluated the influence of the SLCO1B3 rs11045585 polymorphism. This gene encodes a solute carrier organic anion transporter member, which is expressed specifically in the basolateral membrane of hepatocytes and was previously reported to affect toxicity caused by docetaxel [9, 14]. Homozygosity of allele A, G and heterozygosity were observed in 63.6, 2.6 and 33.8 % of the patients, respectively (see Table 2). No influence of the rs11045585 polymorphism on the frequency of leucopenia after the first or entire induction chemotherapy (*p* = 0.925; *p* = 0.99) or grade 3 or 4 infection (*p* = 0.77) was observed.

## Discussion

Despite improvements of supportive care in medical oncology treatment related toxicity remains a major obstacle in patients with advanced head and neck cancer. Therefore, a more precise clarification of the benefits of intensive induction chemotherapy and concomitant drug regimens during RT is needed.

Our cohort of consecutively treated patients represents one of the largest cohorts treated outside a clinical trial with TPF. The clinical data with a median PFS of 20 months, a median OS of 31 months and a treatment related mortality of 3.8 % related to TPF are in line with the published data from clinical trials and retrospective analyses [3, 4, 15–18]. The ORR to induction chemotherapy was high and the vast majority of patients were referred for further RT as planned.

Despite this multimodality treatment is a standard option in patients with advanced head and neck cancer, the role of the concomitant cytotoxic agents after induction chemotherapy is less clear. In combination with postoperative and definitive RT high dose cisplatin therapy is considered as gold standard in feasible patients [19, 20], but a recent phase 2 trial doubted its role in comparison to cetuximab after induction chemotherapy [21]. Because of suggested higher efficacy concomitant cisplatin therapy is considered as treatment standard after induction chemotherapy in our cancer center in fit patients without contraindications for or severe toxicities during induction chemotherapy. This intensive management was associated with a better clinical outcome than treatment with carboplatin or cetuximab. To exclude a bias of patients, who were switched to cetuximab because of a lack of response to cytotoxic induction therapy we also analyzed only the subgroup of patients, who achieved at least a PR after induction therapy. Cisplatin was also associated with a better LRC and PFS than non-intensive therapy, but OS did not reach statistical significance in these patients. Therefore, we continue to use cisplatin as concomitant regimen after induction chemotherapy in patients without contraindications. Nevertheless, we want to point out a selection bias in this analysis due to the fact that only patients in good clinical condition were considered for this intensive treatment with high dose cisplatin during RT.

Besides clinical risk factors such as age or comorbidites, the tolerance and efficacy of cytotoxic drugs may be influenced by SNPs of genes involved in drug metabolism. Polymorphism of the ERCC2 and XPD gene have been described to associated with a better OS in patients treated with various cisplatin based chemotherapy regimens, but only 40 patients were treated with modern induction chemotherapy including taxanes [7]. We tested these SNPS together with polymorphisms of the ERCC1 gene, which are also associated with a better outcome with cisplatin based treatment [13], in our cohort of 78 uniformly treated patients with TPF, but we could not reproduce any association of a certain polymorphism with the clinical outcome.

We further tested the influence of the CYP39A1-rs7761731 and SLCO1B3-rs11045585 polymorphisms on adverse effects during TPF chemotherapy. The addition of docetaxel to cisplatin and 5-fluorouracil nearly doubled the rate of leucopenia in two randomized trials and therefore a genomic marker to identify patients at high risk for leucopenia would be useful. CYP39A1 is a microsomal cytochrome P450 enzyme and is expressed in the liver, where docetaxel is mainly metabolized. It is involved in the conversion of cholesterol to bile acids. There is one report so far published that genotype A of this gene was associated with neutropenia after docetaxel treatment in Japanese patients with gynecological malignancies [8]. We could show now for the first time that Caucasian patients with both genotypes AA and AT are more prone to severe leucopenia in the first cycle of TPF treatment in comparison to patients with TT genotype despite prophylactic growth factor use in all patients. We also observed a higher rate of infections or death during TPF in patients with the genotype AA despite antibiotic prophylaxis. Nevertheless, we could not reproduce the effect of the SLCO1B3 rs11045585 polymorphism on docetaxel-induced leucopenia reported in previous literature [9–11]. It remains unclear, if this effect was diminished by the use of prophylactic growth factor or the combined effect of 3 cytotoxic drugs.

Our work has several limitations. Despite the fact that we provided a detailed follow-up of all consecutively treated patients this analysis may imply an operative selection bias. The size of our cohort is larger than most of the published retrospective cohorts of patients treated with TPF [15–18], but of course cannot be compared with large phase 3 trials [3, 4]. Absolute neutrophil counts in addition to leukocytes counts would have been desirable, but were not available for the majority of patients, because the total leukocyte count is often completely sufficient in clinical practice. Nevertheless, documentation of toxicity was precise, especially in the first cycle of treatment because of hospitalization of all patients until bone marrow recovery with routine blood count testing at least every other day. Assessment of the HPV status was not available in this analysis, but this has no proven value for the choice of induction chemotherapy during clinical routine.

Furthermore, our results suggest a superiority of the use of concomitant cisplatin in patients already treated with TPF for subsequent radiochemotherapy. Although we could show this effect also in patients with a radiological response to TPF, these results may be biased due to less comorbdities and better performance status in patients further treated with cisplatin. Therefore, we think this question will only be clarified in a prospective randomized clinical trial.

## Conclusion

In summary, we could show the feasibility of intensive induction chemotherapy using the TPF regimen in a large single center cohort, but significant toxicity despite growth factor and antibiotic support remains a major concern in these patients. This is the first report of a gene polymorphism associated with treatment related toxicity during induction chemotherapy in patients with head and neck cancer. This finding is hypothesis-generating and further evaluation in other larger cohorts is needed to confirm this observation.

## Abbreviations

RT: Radiotherapy
RCTX: Radiochemotherapy
OS: Overall survival
TPF: Chemotherapy with a platinum compound, docetaxel and 5-fluorouracill
LCR: Loco regional control
OS: Overall survival
PFS: Progression free survival
SNP: Single nucleotide polymorphism
HIV: Human immunodeficiency virus
AJCC: American Joint Committee on Cancer
ORR: Overall response rate

## References

[1] Argiris A, Karamouzis MV, Raben D, Ferris RL (2008). Head and neck cancer. Lancet.

[2] Pignon JP, le Maitre A, Maillard E, Bourhis J (2009). Meta-analysis of chemotherapy in head and neck cancer (MACH-NC): an update on 93 randomised trials and 17,346 patients. Radiother Oncol.

[3] Posner MR, Hershock DM, Blajman CR, Mickiewicz E, Winquist E, Gorbounova V (2007). Cisplatin and fluorouracil alone or with docetaxel in head and neck cancer. N Engl J Med.

[4] Vermorken JB, Remenar E, van Herpen C, Gorlia T, Mesia R, Degardin M (2007). Cisplatin, fluorouracil, and docetaxel in unresectable head and neck cancer. N Engl J Med.

[5] Haddad R, O’Neill A, Rabinowits G, Tishler R, Khuri F, Adkins D (2013). Induction chemotherapy followed by concurrent chemoradiotherapy (sequential chemoradiotherapy) versus concurrent chemoradiotherapy alone in locally advanced head and neck cancer (PARADIGM): a randomised phase 3 trial. Lancet Oncol.

[6] Zhong LP, Zhang CP, Ren GX, Guo W, William WN, Sun J (2013). Randomized phase III trial of induction chemotherapy with docetaxel, cisplatin, and fluorouracil followed by surgery versus up-front surgery in locally advanced resectable oral squamous cell carcinoma. J Clin Oncol.

[7] Quintela-Fandino M, Hitt R, Medina PP, Gamarra S, Manso L, Cortes-Funes H (2006). DNA-repair gene polymorphisms predict favorable clinical outcome among patients with advanced squamous cell carcinoma of the head and neck treated with cisplatin-based induction chemotherapy. J Clin Oncol.

[8] Uchiyama T, Kanno H, Ishitani K, Fujii H, Ohta H, Matsui H (2012). An SNP in CYP39A1 is associated with severe neutropenia induced by docetaxel. Cancer Chemother Pharmacol.

[9] Choi JR, Kim JO, Kang DR, Shin JY, Zhang XH, Oh JE (2015). Genetic Variations of Drug Transporters Can Influence on Drug Response in Patients Treated with Docetaxel Chemotherapy. Cancer Res Treat.

[10] Chew SC, Singh O, Chen X, Ramasamy RD, Kulkarni T, Lee EJ (2011). The effects of CYP3A4, CYP3A5, ABCB1, ABCC2, ABCG2 and SLCO1B3 single nucleotide polymorphisms on the pharmacokinetics and pharmacodynamics of docetaxel in nasopharyngeal carcinoma patients. Cancer Chemother Pharmacol.

[11] Kiyotani K, Mushiroda T, Kubo M, Zembutsu H, Sugiyama Y, Nakamura Y (2008). Association of genetic polymorphisms in SLCO1B3 and ABCC2 with docetaxel-induced leukopenia. Cancer Sci.

[12] Bender R, Lange S (2001). Adjusting for multiple testing--when and how?. J Clin Epidemiol.

[13] Isla D, Sarries C, Rosell R, Alonso G, Domine M, Taron M (2004). Single nucleotide polymorphisms and outcome in docetaxel-cisplatin-treated advanced non-small-cell lung cancer. Ann Oncol.

[14] Konig J, Cui Y, Nies AT, Keppler D (2000). Localization and genomic organization of a new hepatocellular organic anion transporting polypeptide. J Biol Chem.

[15] Billan S, Kaidar-Person O, Atrash F, Doweck I, Haim N, Kuten A (2013). Toxicity of induction chemotherapy with docetaxel, cisplatin and 5-fluorouracil for advanced head and neck cancer. Isr Med Assoc J.

[16] Rampino M, Bacigalupo A, Russi E, Schena M, Lastrucci L, Iotti C (2012). Efficacy and feasibility of induction chemotherapy and radiotherapy plus cetuximab in head and neck cancer. Anticancer Res.

[17] Ko EC, Genden EM, Misiukiewicz K, Som PM, Kostakoglu L, Chen CT (2012). Toxicity profile and clinical outcomes in locally advanced head and neck cancer patients treated with induction chemotherapy prior to concurrent chemoradiation. Oncol Rep.

[18] Kim B, Dillman RO, Chen P, Hafer R, Cox C, Barth N (2012). A retrospective study of induction chemotherapy with docetaxel, cisplatinum, and 5-fluorouracil followed by concurrent radiotherapy with cetuximab in locally advanced head and neck cancer. Am J Otolaryngol.

[19] Cooper JS, Pajak TF, Forastiere AA, Jacobs J, Campbell BH, Saxman SB (2004). Postoperative concurrent radiotherapy and chemotherapy for high-risk squamous-cell carcinoma of the head and neck. N Engl J Med.

[20] Bernier J, Domenge C, Ozsahin M, Matuszewska K, Lefebvre JL, Greiner RH (2004). Postoperative irradiation with or without concomitant chemotherapy for locally advanced head and neck cancer. N Engl J Med.

[21] Lefebvre JL, Pointreau Y, Rolland F, Alfonsi M, Baudoux A, Sire C (2013). Induction chemotherapy followed by either chemoradiotherapy or bioradiotherapy for larynx preservation: the TREMPLIN randomized phase II study. J Clin Oncol.

